# Supplementary material for: Cathepsin B-Cleavable Polymeric Photosensitizer Prodrug for Selective Photodynamic Therapy: In Vitro Studies
Source: Pharmaceuticals (Basel). 2022 Apr 30;15(5):564. doi: 10.3390/ph15050564 (PMC9146285; doi:10.3390/ph15050564)
Supplement: Supplementary file 1 [file pharmaceuticals-15-00564-s001.zip › pharmaceuticals-1689989-supplementary.pdf]

pharmaceuticals

Article

Cathepsin B cleavable-polymeric photosensitizer prodrug for selective photodynamic therapy: *In Vitro* Studies

Manish Jain [1,5], Jordan Bouilloux [2], Ines Borrego [1], Stéphane Cook [1,6], Hubert van den Bergh [3], Norbert Lange [2], Georges Wagnieres [4], Marie-Noelle Giraud [1*]

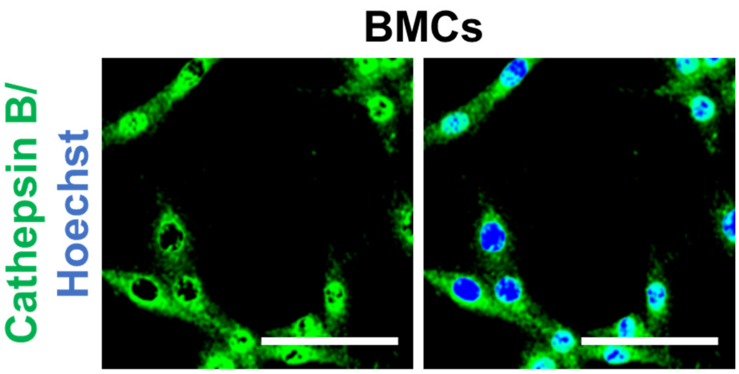

Supplementary Figure S1: Expression of Cathepsin B in BMCs. Representative images showing immunostaining for Cathepsin B (red) in BMCs isolated from rat bone marrow. Nuclei are counterstained with Hoechst (blue). Scale bars: 100 µm.

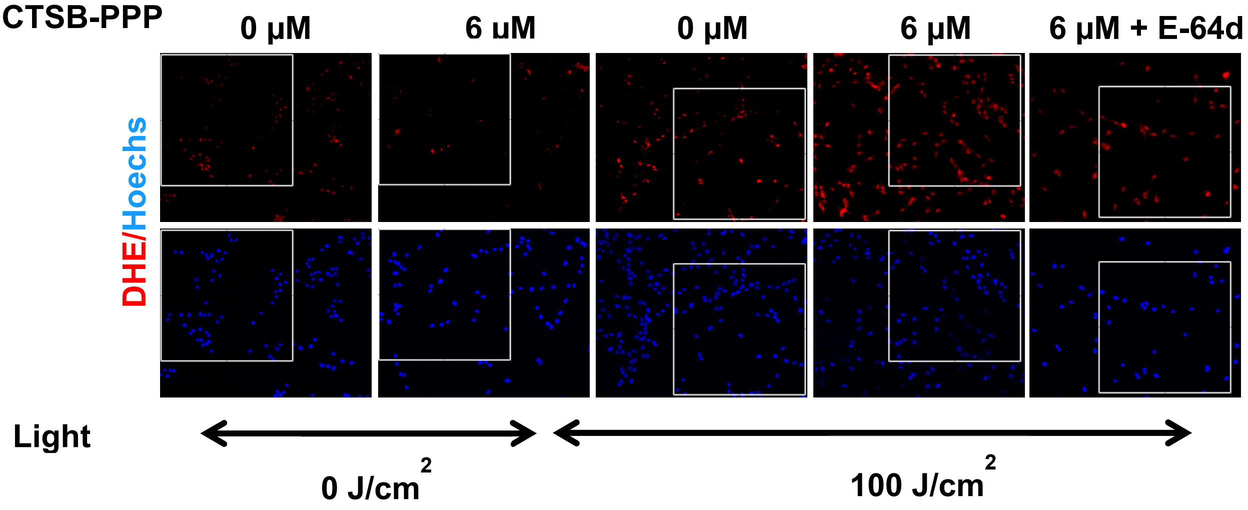

Supplementary Figure S2. (A) **Original** fluorescent photomicrograph of DHE (red; Upper panel and Hoechst staining (blue; bottom panel)

Supplementary Table S1- Related analysis for Figure 3A

Time	0 Min	10 Min	20 Min	60 Min	24 h
CTSB-PPP Fluorescence Intensity	304.876<br>234.5927<br>295.0668	1453.019<br>2444.654<br>1899.411	2100.603<br>2466.619<br>2064.937	3153.928<br>3460.447<br>4140.803	2762.526<br>3281.686<br>3090.593

Supplementary Table S2- Related analysis for Figure 4B

Time	0 Min	10 Min	20 Min	60 Min	24 h
CTSB-PPP 6 μM	3053.079	85468.01	78170.87	184230	44379.67
Light Dose (100 J/cm2)	46756.5	22664.46	17795.49	182681	90766.75
E-64d (50 μM)	17301.85	24167.12	42616.14	240514	124910.3

Supplementary Table S3- Related analysis for Figure 7

CTSB-PPP 0 uM	CTSB-PPP 3 uM	CTSB-PPP 6 uM	CTSB-PPP 12 uM	CTSB-PPP 24 uM	CTSB-PPP 48 uM	CTSB-PPP 6 uM + E-64d (50 μM	CTSB-PPP 12 uM + E-64d (50 μM
304.876	1352.165	4187.510	1713.500	4286.285	2987.563	6707.696	7909.174
234.593	1326.665	3557.992	2518.688	3855.739	2865.938	7849.319	7992.087
295.067	951.514	4162.107	2727.938	5447.929	3201.813	5965.570	5803.124
		4787.583	2544.125	5745.100	2165.125		
			1064.313		2964.375		
